# MnCo_2_O_4_ spinel nanoparticles as a dusting powder for sebaceous and natural latent fingermarks on substrates of different porosities

**DOI:** 10.1016/j.fsisyn.2026.100754

**Published:** 2026-11-07

**Authors:** Shivam Kumar Mittal, Kumud Kant Awasthi, Vaibhav Sharma, Garima Awasthi, Rachit Manchanda, Gaurav Kumar Singh, Yogesh Kumar, Harsh Pandey, Mahipal Singh Sankhla

**Affiliations:** a School of Basic and Applied Sciences, K. R. Mangalam University, Gurugram, Haryana, India; b Department of Medical Elementology and Toxicology, School of Chemical and Life Sciences, Jamia Hamdard, New Delhi, India; c Department of Computer Science Engineering, University Institute of Engineering, Chandigarh University, Mohali, Punjab, India; d Department of Forensic Science, Sharda School of Allied Health Sciences, Sharda University, Greater Noida, Uttar Pradesh, India; e Department of Forensic Science, College of Allied and Healthcare Sciences, Teerthanker Mahaveer University, Moradabad, Uttar Pradesh, India; f Department of Biotechnology and Chemical Engineering, Manipal University Jaipur, Rajasthan, India

**Keywords:** Latent fingermark, MnCo_2_O_4_ nanoparticles, Powder dusting, Spinel oxide

## Abstract

Dark particulate developers remain the most widely applied means of visualizing latent fingermarks. Manganese cobalt oxide (MnCo_2_O_4_) nanoparticles were prepared by alkaline coprecipitation at 80 °C and characterized by XRD, FTIR, FESEM, and EDS, which indicated the formation of the cubic spinel phase of MnCo2O4 with a crystallite size of approximately 21 nm, a particle size of approximately 50 nm, and particle aggregation. Sebaceous and natural fingermarks on ten substrates were developed by powder dusting, visually graded, and scored using NIST Fingerprint Image Quality 2 (NFIQ 2). Sebaceous marks received grades of 4 or 5 on eight substrates, whereas natural marks achieved these grades on five substrates. NFIQ 2 scores correlated with visual grades (Spearman ρ = 0.69) and were higher for sebaceous marks (median 33.5 versus 15.0). Substrate texture inflated the automated minutiae counts, and microscopy showed particle deposition largely confined to the ridges.

## 1 Introduction

Friction ridge impressions remain among the most frequently recovered forms of physical evidence because the arrangement of ridge endings, bifurcations, and other minutiae permits comparison with reference prints [1]. Most marks encountered at scenes are latent: a thin residue transferred from the fingertip to the contacted surface that is invisible or barely visible under ordinary illumination and must be developed before it can be examined [1], [2]. The reliability of that development step directly limits the extent to which ridge detail is conveyed to the examiner. In this paper, fingermark (shortened to mark) denotes the trace deposited on a surface, before or after development. In contrast, fingerprint is reserved for reference prints and for established product and software names.

Latent fingermark residue contains material from three sources. Eccrine sweat contains water, inorganic salts, amino acids, and other low-molecular-weight compounds. Sebaceous secretion, transferred to the fingertips through contact with the face and scalp, contains fatty acids, wax esters, glycerides, cholesterol, and squalene. Exogenous material is acquired from surfaces and products touched before deposition [3], [4]. The proportions of these constituents differ between donors and between deposits from the same donor, and the residue changes physically and chemically after deposition [5]. A natural, ungroomed mark is a variable mixture of eccrine and sebaceous material whose lipid content depends on recent contact of the fingers with the face or hair. In contrast, a groomed sebaceous mark is deliberately enriched in sebaceous lipids [3], [5]. A developer must interact with a deposit whose quantity and composition are unknown in advance, on a substrate whose porosity, texture, and color vary from case to case.

Powder dusting remains the most widely applied technique for nonporous surfaces because it is rapid, inexpensive, and requires minimal equipment [2], [6]. Fine particles adhere mechanically and adhesively to the moist or greasy ridge deposit, thereby contrasting against the background. Their performance depends on particle size, shape, surface chemistry, and color: particles that are too coarse adhere poorly to the ridge residue, whereas particles with excessive affinity for the substrate cause background staining that obscures minutiae [6], [7]. These constraints have motivated the evaluation of engineered particulate developers in which composition and morphology can be controlled at the nanoscale.

Nanomaterials evaluated for fingermark detection include metal and metal oxide nanoparticles, semiconductor quantum dots, carbon dots and functionalized silica, selected for their dimensions, tunable surface chemistry and, in certain systems, optical or magnetic properties [8], [9], [10], [11], [12], [13], [14], [15]. Among inorganic oxides, ZnO, ZnO–SiO_2_ and TiO_2_ nanopowders have developed marks on a range of surfaces [16], [17], [18], [19], and a comparison of metal and metal oxide nanoparticles showed that their performance depends strongly on the substrate [20]; iron oxide and other magnetic nanoparticles have been applied as magnetic powders that reduce brush contact with the deposit [21], [22], [23]. In parallel, inexpensive powders prepared from plant and waste materials, including gram flour, rose and hibiscus petals, jamun seed, onion, pomegranate peel, aloe vera, water chestnut flour, wheatgrass and neem nanobiochar, have been evaluated on porous and nonporous substrates [24], [25], [26], [27], [28], [29], [30], [31], [32], [33]. A black magnetic powder of cobalt ferrite (CoFe_2_O_4_) has also been reported, indicating that dark spinel ferrites can serve as fingermark developers [34]. Reviews in this field emphasize that nanoscale dimensions alone do not guarantee performance and that aggregation, surface chemistry, substrate compatibility, and application procedures must be examined for each material [9], [10].

Manganese cobalt oxide (MnCo_2_O_4_) is a mixed transition metal oxide that crystallizes in the spinel structure. Cubic MnCo_2_O_4_ nanocrystals can be obtained from aqueous Mn(II) and Co(II) precursors by precipitation and oxidation at low temperature, and the phase and Mn:Co ratio of the product can be adjusted through the synthesis conditions [35], [36]. The material has been examined mainly as an electrocatalyst, an electrochemical sensor modifier, and an electrode material [37], [38], [39], [40], including Mg-doped MnCo_2_O_4_ nanoceramics and MnCo_2_O_4_–exfoliated graphite composites for high-energy-density supercapacitors [41], [42]. Related Mn–Co oxides, such as the double perovskite Bi_2_MnCoO_6_, have been developed as electrodes for asymmetric supercapacitors and solid oxide fuel cells operating at intermediate temperatures [43], [44]. The black color, chemical stability, and accessibility of MnCo_2_O_4_ by a simple coprecipitation route make it a reasonable candidate for a dark dusting powder. The presence of a spinel phase or of nanoscale particles does not, by itself, establish selective adhesion to fingermark residue, and the forensic value of the material has to be determined experimentally on relevant substrates and deposit types.

The available literature on MnCo_2_O_4_ as a latent fingermark developer appears limited. In the present study, MnCo_2_O_4_ nanoparticles were synthesized via alkaline coprecipitation and characterized by X-ray diffraction (XRD), Fourier transform infrared (FTIR) spectroscopy, field-emission scanning electron microscopy (FESEM), and energy-dispersive X-ray spectroscopy (EDS). The powder was then applied to sebaceous and natural fingermarks deposited on ten substrates of differing porosity. Developed marks were graded visually for clarity and, to reduce dependence on observer judgment, were also scored with the NIST Fingerprint Image Quality 2 (NFIQ 2) algorithm, which returns a quality score and native quality measures, including the number of minutiae detected by an automated extractor [45], [46]. Deposition of the powder at the level of individual ridges was compared microscopically with that of a commercial black fingerprint powder. The aim was to relate the structural and morphological characteristics of the powder to its development performance and to identify the substrates and deposit types for which the material is suitable at the laboratory feasibility stage.

## 2 Materials and methods

### 2.1 Chemicals

Manganese(II) chloride tetrahydrate (MnCl_2_·4H_2_O; SRL), cobalt(II) nitrate hexahydrate (Co(NO_3_)_2_·6H_2_O; HiMedia) and sodium hydroxide (NaOH; Qualigens) were used as received. Deionized (DI) water was used as the solvent for all solutions and dispersions, and ethanol was used for washing the product. Sirchie black fingerprint powder (Sirchie, USA) served as the commercial reference powder.

### 2.2 Synthesis of MnCo_2_O_4_ nanoparticles

MnCo_2_O_4_ nanoparticles were prepared by coprecipitation (Fig. 1). MnCl_2_·4H_2_O (1 mmol, 0.198 g) and Co(NO_3_)_2_·6H_2_O (2 mmol, 0.582 g), corresponding to the Mn:Co molar ratio of 1:2 required for MnCo_2_O_4_, were dissolved together in DI water, and the solution was ultrasonicated for 15 min and then stirred magnetically at 500 rpm for 30 min. Aqueous NaOH (2 M), prepared separately in DI water, was added dropwise under continuous stirring until the pH reached 11. The mixture was heated at 80 °C for 3 h under continuous stirring and allowed to cool naturally to room temperature. The precipitate was washed three times with ethanol and DI water, dried at 60 °C for 12 h, and ground to a fine powder.

**Fig. 1:**
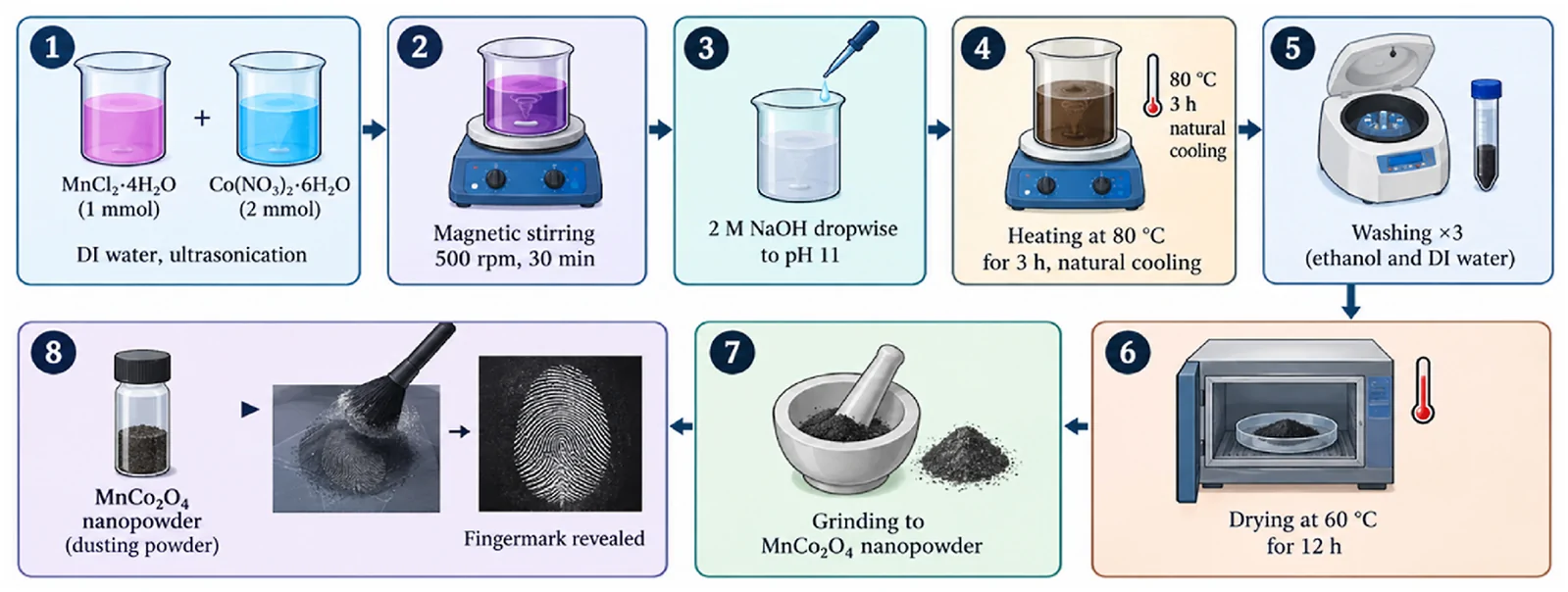
Coprecipitation route for the synthesis of MnCo_2_O_4_ nanoparticles and their application as a fingermark dusting powder: (1) dissolution of 1 mmol MnCl_2_·4H_2_O and 2 mmol Co(NO_3_)_2_·6H_2_O in DI water with ultrasonication; (2) magnetic stirring at 500 rpm for 30 min; (3) dropwise addition of 2 M NaOH to pH 11; (4) heating at 80 °C for 3 h followed by natural cooling; (5) washing three times with ethanol and DI water; (6) drying at 60 °C for 12 h; (7) grinding; (8) powder dusting of latent fingermarks.

Under these conditions, Mn(II) and Co(II) precipitate as hydroxides at pH 11, and the mixed hydroxide is oxidized by dissolved and atmospheric oxygen during heating to form the spinel oxide, in which the average cation oxidation state rises from +2 to +8/3 [35], [36]. The overall stoichiometry can be represented as follows:

(1) Mn^2+^ + 2OH^−^ → Mn(OH)_2_

(2) Co^2+^ + 2OH^−^ → Co(OH)_2_

(3) Mn(OH)_2_ + 2Co(OH)_2_ + ½O_2_ → MnCo_2_O_4_ + 3H_2_O

(4) MnCl_2_ + 2Co(NO_3_)_2_ + 6NaOH + ½O_2_ → MnCo_2_O_4_ + 2NaCl + 4NaNO_3_ + 3H_2_O

Eqs. (1), (2), (3) are balanced in mass and charge, and Eq. (4) gives the corresponding overall reaction of the precursor salts, in which NaCl and NaNO_3_ remain in solution and are removed by washing. The equations describe the overall stoichiometry; intermediate species, such as partially oxidized oxyhydroxides, were not characterized.

### 2.3 Characterization

The crystalline structure of the powder was examined by XRD using Cu Kα radiation over a 2θ range of 20° to 70°. The average crystallite size (*D*) was estimated from the broadening of the diffraction reflections using the Debye–Scherrer equation, *D* = *K*λ/(β cos θ), where *K* is the shape factor (0.9), λ is the X-ray wavelength (0.154 nm), β is the full width at half maximum of the reflection in radians, and θ is the Bragg angle [47], [48]. Phases were identified by comparison of the observed reflections with the reference pattern of cubic spinel MnCo_2_O_4_ (JCPDS card No. 23-1237) [39]. Reflection positions were read from the diffraction pattern with a resolution of approximately 0.1° 2θ; interplanar spacings (*d*) were calculated from Bragg's law, and the cubic lattice parameter was calculated as *a* = *d*(*h*^2^ + *k*^2^ + *l*^2^)^1/2^ [48]. FTIR spectra were recorded between 4000 and 400 cm^−1^ to identify surface functional groups and metal–oxygen vibrations. Particle morphology was examined by FESEM (Zeiss; InLens detector, accelerating voltage 20 kV, working distance 7.0 mm) at magnifications of 100 000× and 250 000×, and elemental composition was determined by EDS.

### 2.4 Fingermark donors and deposition

Fingermarks were collected from 120 donors of both sexes, aged 18–30 years. Two deposit types were prepared. For sebaceous marks, donors gently rubbed their fingertips across sebum-rich areas, such as the forehead or the side of the nose, for approximately 5–10 s before deposition. Natural marks were deposited without deliberate enrichment or depletion of eccrine or sebaceous material, and represented the normal, unguided deposit of each donor, in line with the recommendation that natural marks be used to avoid the overestimation of performance associated with groomed marks [49], [50]. Donors acclimatized to the laboratory environment for approximately 15–20 min and were instructed not to apply hand creams, lotions, sanitizers, or other topical products before sampling. Marks were deposited with the index finger onto clean, labeled substrates for approximately 5 s under moderate, comfortable pressure, without pressing, rolling, or dragging the finger. The same researcher supervised all depositions wherever possible. Substrates were handled with clean gloves and stored under laboratory conditions until development, which was carried out without a deliberate aging interval.

All participants were informed of the study's purpose and procedures, and written informed consent was obtained before sample collection. The study followed the applicable institutional ethical guidelines for research involving human participants. No personally identifying information was linked to the fingermark samples, and each donor was assigned a unique study code.

### 2.5 Substrates

Ten substrates representing surfaces commonly encountered at crime scenes were selected and grouped by porosity (Table 1): iron, steel, aluminum foil, ceramic tile, glass, plastic and rubber (nonporous), leather (semiporous), and cardboard and white paper (porous). Substrates were cleaned with acetone before deposition to remove existing contamination.

**Table 1:** Substrates used for fingermark development, porosity class, and corresponding panel in Fig. 4, Fig. 5.

Substrate	Porosity class
Iron	Nonporous
Steel	Nonporous
Aluminum foil	Nonporous
Ceramic tile	Nonporous
Glass	Nonporous
Plastic	Nonporous
Rubber	Nonporous
Leather	Semiporous
Cardboard	Porous
White paper	Porous

### 2.6 Development and imaging

Latent marks were developed by conventional powder dusting. A small quantity of MnCo_2_O_4_ powder was applied to the marked area with a camel hair brush in light circular strokes, and excess powder was removed by gentle brushing. Developed marks were photographed with a Samsung Galaxy S23 smartphone camera under controlled laboratory illumination, with the camera positioned perpendicular to the substrate at a consistent distance. Focus and imaging conditions were kept as constant as possible so that the complete ridge pattern was captured for every mark.

### 2.7 Objective image quality assessment with NFIQ 2

Each developed mark was cropped from its original photograph to the mark's area and converted to 8-bit grayscale using luminance weighting. Image processing was performed in Python using scikit-image [51]. NFIQ 2 is calibrated for images at 500 pixels per inch (ppi) [45], whereas the photographs carried no scale. The dominant ridge period of each crop was estimated from the two-dimensional Fourier spectra of overlapping 48 × 48 pixel blocks (Hann window) within the mark, selecting the spectral peak in the band corresponding to periods of 3 to 16 pixels and taking the weighted median across blocks with a peak-to-mean ratio above 4. Each crop was then resampled by bicubic interpolation so that the ridge period was 9 pixels, equivalent to a ridge-to-ridge distance of approximately 0.46 mm at 500 ppi and consistent with adult fingerprint ridge densities of approximately 11 to 13 ridges per 25 mm^2^ [52]. No dominant ridge frequency was detected for the natural mark on the leather, so the median scale factor from the remaining images was applied to it. A global linear contrast stretch between the 1st and 99th percentiles of intensity was applied to all images; no other enhancement was performed.

The normalized images were analyzed using NFIQ 2 version 2.3.1, the NIST reference implementation of ISO/IEC 29794-4, which returns a unified quality score from 0 to 100, along with native quality measures [45]. The measures reported here are the local clarity score (LCS), orientation certainty level (OCL), orientation flow (OF), frequency domain analysis (FDA), and ridge valley uniformity (RVUP), each summarized by its mean over image blocks, and the number of minutiae detected by the FingerJet FX OSE extractor embedded in NFIQ 2. Minutiae locations and qualities were obtained from the same extractor as ISO/IEC 19794-2 templates for visualization.

### 2.8 Microscopic comparison with a commercial powder

To examine particle distribution at the ridge level, marks deposited on glass were developed with MnCo_2_O_4_ powder and, separately, with Sirchie black fingerprint powder under the same brushing procedure. The developed ridges were examined under an optical microscope in transmitted light and photographed through the eyepiece.

### 2.9 Grading of developed marks and data analysis

Development quality was assessed on a five-point clarity scale (Table 2) that considers ridge continuity, visibility of minutiae, and background interference, the parameters used in absolute grading schemes for fingermark research [49], [50]. Each mark was graded independently by two assessors, and disagreements were resolved by consensus. Marks developed on the same substrate showed consistent development across donors, and one representative mark was randomly selected for each substrate and deposit type. These marks are shown in Fig. 4, Fig. 5, yielding 20 graded substrate-deposit combinations (Table 4).

**Table 2:** Five-point clarity scale used to grade developed fingermarks.

Grade	Descriptor	Ridge detail criterion
5	Excellent	Continuous ridge flow over the whole mark; minutiae and pattern area clearly resolved; negligible background deposition
4	Good	Continuous ridge flow over most of the mark; minutiae identifiable; minor discontinuity or background deposition
3	Moderate	Ridge flow visible but interrupted by smudging, uneven deposition or background interference; minutiae resolvable only in parts of the mark
2	Poor	Faint or partial ridge development; ridge continuity largely lost; minutiae not reliably resolvable
1	No useful development	No ridge detail or only isolated ridge fragments

**Table 3:** Comparison of the XRD reflections of the synthesized powder with the reference pattern of cubic spinel MnCo_2_O_4_ (JCPDS card No. 23-1237).

*hkl*	2θ observed (°)	*d* observed (Å)	2θ JCPDS 23-1237 (°)	*d* JCPDS 23-1237 (Å)	Δ2θ (°)
(220)	31.0	2.882	30.54	2.925	0.5
(311)	36.4	2.466	35.995	2.493	0.4
(222)^a^	38.4	2.342	37.66^b^	2.387	0.7
(400)	44.5	2.034	43.759	2.067	0.7
(422)^a^	55.4	1.657	54.336	1.687	1.1
(511)	58.9	1.567	57.909	1.591	1.0
(440)	64.5	1.444	63.622	1.461	0.9

**Table 4:** Clarity grades of sebaceous and natural fingermarks developed with MnCo_2_O_4_ powder.

Substrate	Porosity class	Sebaceous	Natural	Difference
Iron	Nonporous	5	4	1
Steel	Nonporous	5	3	2
Aluminum foil	Nonporous	4	4	0
Ceramic tile	Nonporous	5	5	0
Glass	Nonporous	3	3	0
Plastic	Nonporous	4	2	2
Rubber	Nonporous	4	2	2
Leather	Semiporous	3	2	1
Cardboard	Porous	5	5	0
White paper	Porous	5	4	1
Mean (median)		4.3 (4.5)	3.4 (3.5)	0.9

Grades and NFIQ 2 scores were summarized as mean and median values by deposit type and porosity class. Because grades are ordinal and the two deposit types were applied to the same substrates, sebaceous and natural marks were compared using the Wilcoxon signed-rank test, paired by substrate (two-sided, α = 0.05), excluding pairs with equal values according to the standard procedure. Agreement between visual grades and NFIQ 2 outputs was assessed with Spearman rank correlation. An image for which NFIQ 2 could not compute a score was assigned the lowest value (0) in rank-based tests and excluded from means. Analyses were performed with SciPy 1.18.1 in Python.

## 3 Results

### 3.1 Structure and surface chemistry of MnCo_2_O_4_

The XRD pattern of the synthesized powder (Fig. 2a) shows diffraction maxima superimposed on a broad, sloping background. The reflections were indexed to the (220), (311), (222), (400), (422), (511), and (440) planes of cubic spinel MnCo_2_O_4_ by comparison with JCPDS card No. 23-1237 (Table 3), with the (311) reflection being the most intense [38], [39]. The low intensity of the reflections relative to the background and their breadth indicate a nanocrystalline product with limited long-range order. The average crystallite size, calculated using the Debye–Scherrer equation, was approximately 21 nm.

**Fig. 2:**
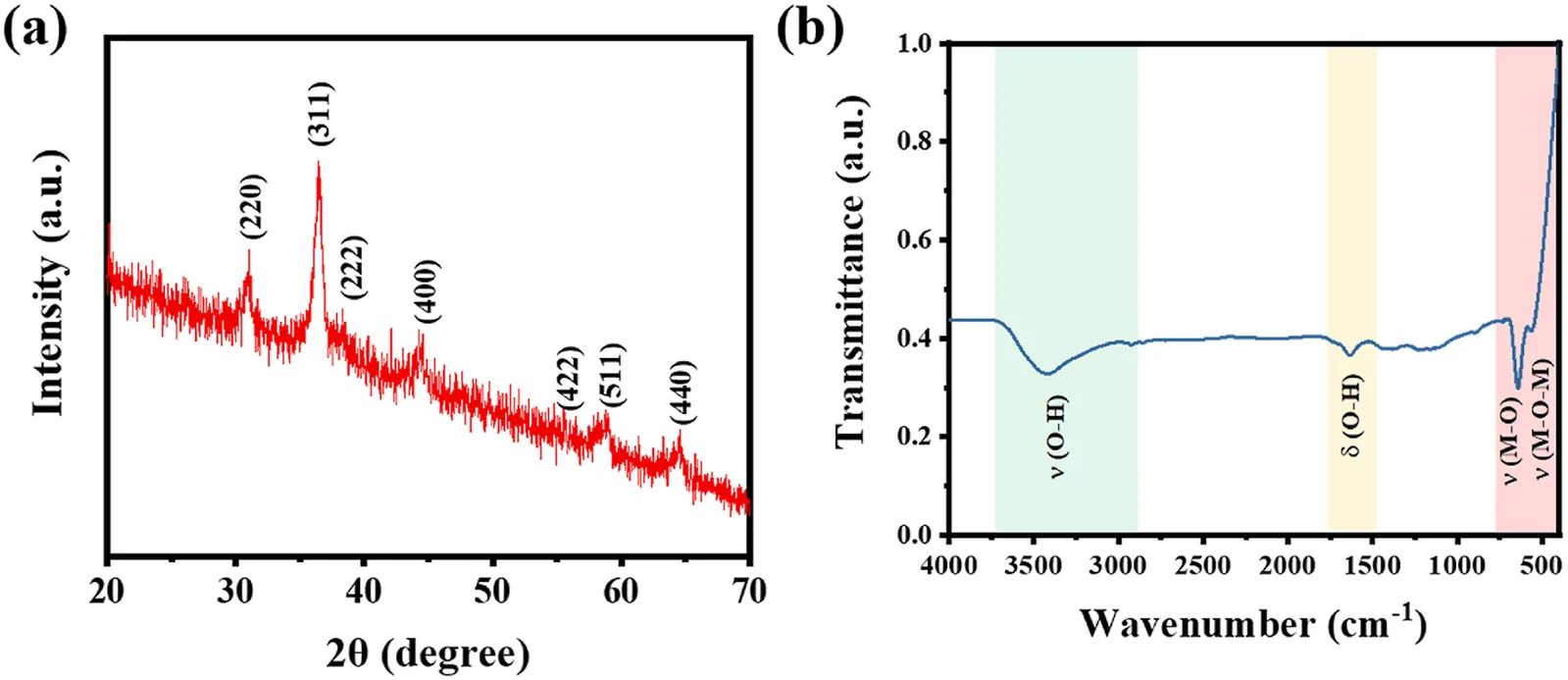
(a) XRD pattern with reflections indexed to cubic spinel MnCo_2_O_4_ (JCPDS card No. 23-1237) and (b) FTIR spectrum of the synthesized MnCo_2_O_4_ nanoparticles.

The FTIR spectrum (Fig. 2b) contains a broad band in the 3700–2900 cm^−1^ region assigned to O–H stretching of surface hydroxyl groups and adsorbed water, and a band in the 1770–1480 cm^−1^ region assigned to the H–O–H bending vibration of adsorbed water, labeled δ(O–H) in Fig. 2b. The strongest absorption lies below 760 cm^−1^, where two bands are assigned to metal–oxygen stretching vibrations, ν(M − O) and ν(M–O–M), with M representing Mn or Co. In spinel oxides, the higher frequency band in this region is associated with metal–oxygen vibrations at tetrahedral sites and the lower frequency band with octahedral sites [39], [53]. The presence of these bands supports the formation of the metal-oxide framework identified by XRD.

### 3.2 Morphology and elemental composition

The FESEM micrograph at 100 000× (Fig. 3a) shows a dense layer of fine, predominantly quasi-spherical particles that tend to form clusters of varying sizes. At 250 000× (Fig. 3b), the clusters are resolved into smaller granular units with an irregular surface texture, and interparticle voids are visible between aggregates. The average particle size of approximately 50 nm was estimated from FESEM images by measuring the dimensions of more than 100 individual particles. This value exceeds the XRD crystallite size of approximately 21 nm, which is expected because an individual particle can contain more than one coherently diffracting domain, and aggregation increases the apparent dimensions of observed features [48].

**Fig. 3:**
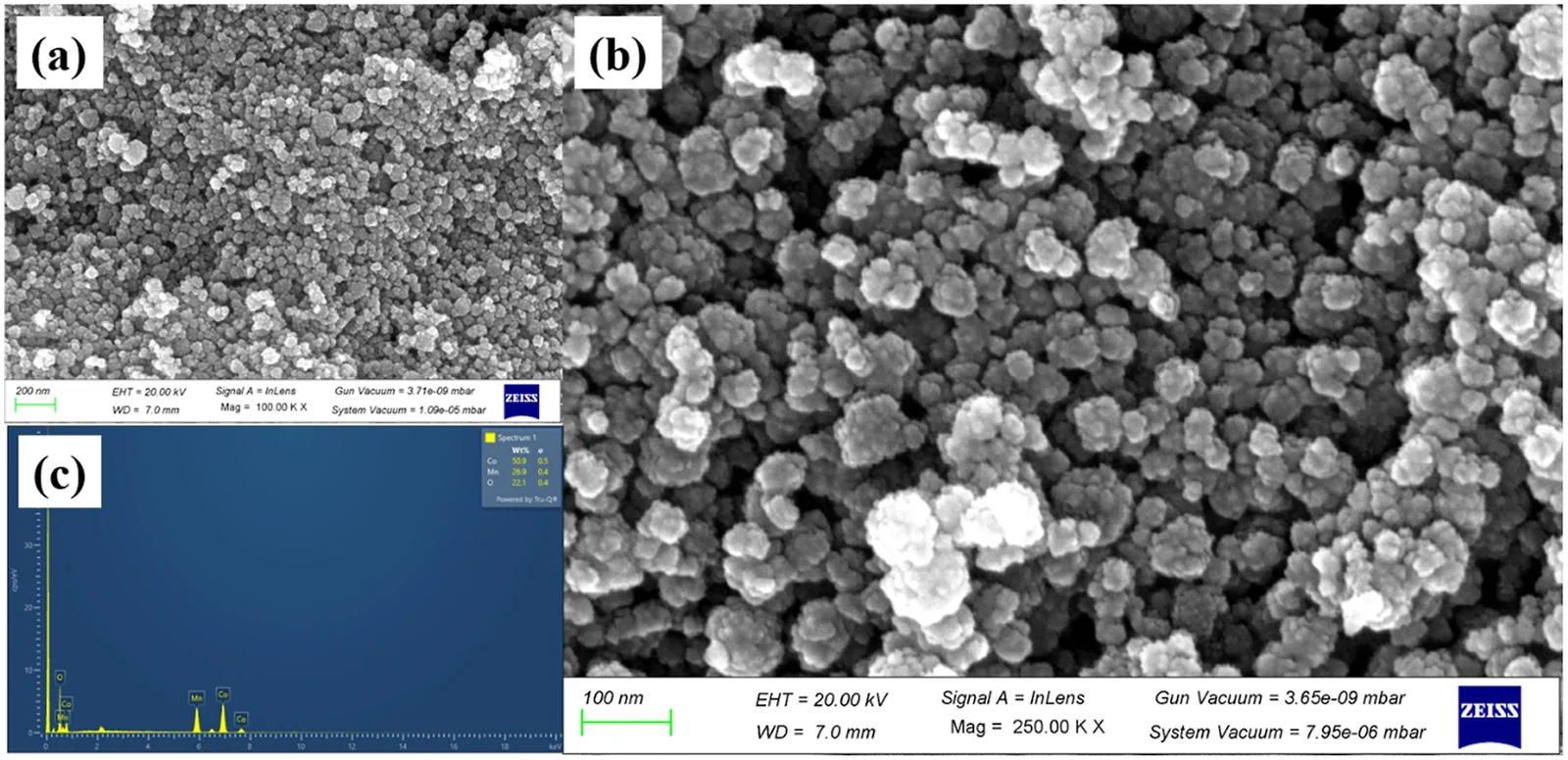
FESEM micrographs of MnCo_2_O_4_ nanoparticles at (a) 100 000× and (b) 250 000× magnification, and (c) the corresponding EDS spectrum.

**Fig. 4:**
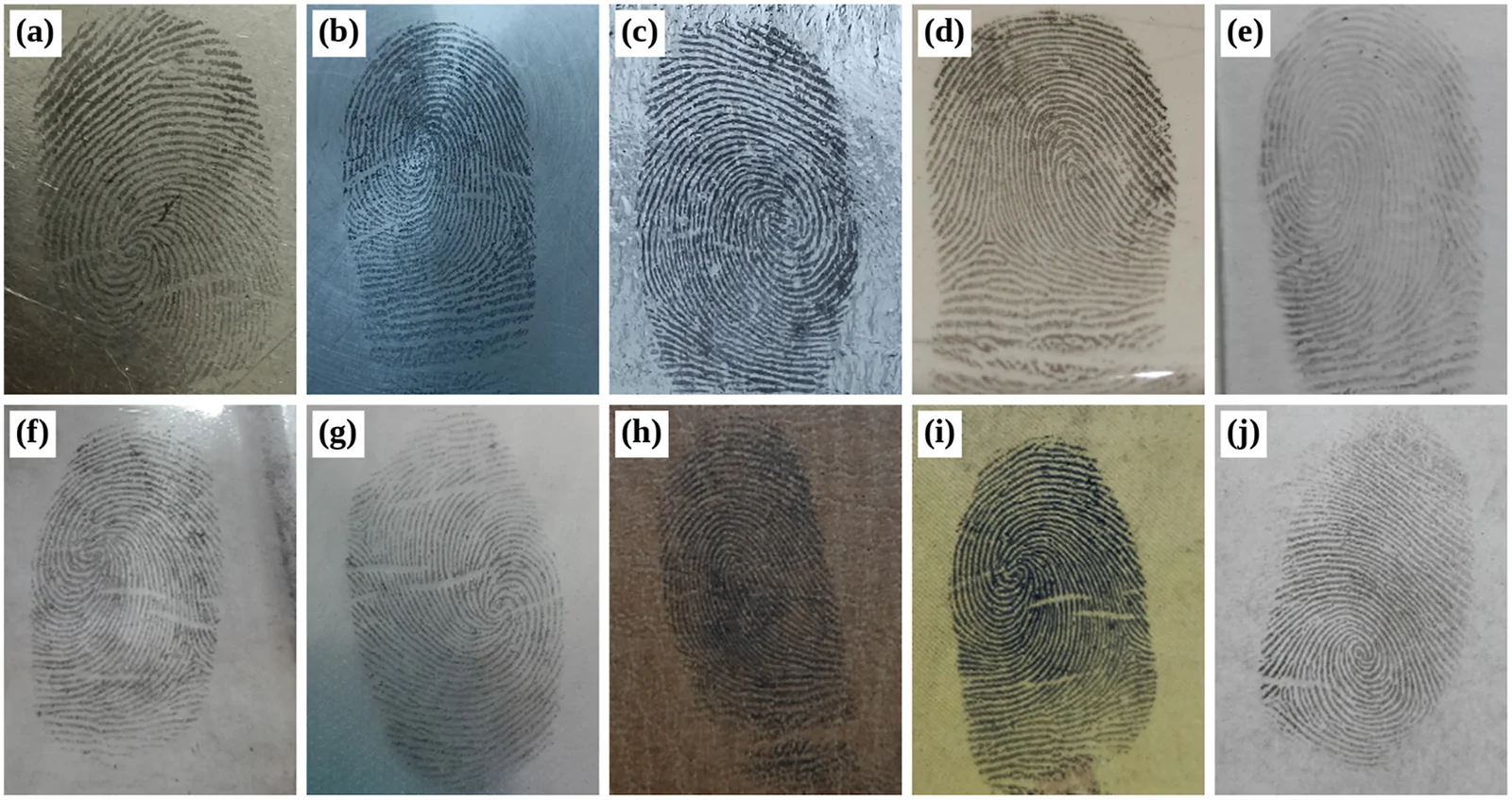
Sebaceous fingermarks developed with MnCo_2_O_4_ powder on (a) iron, (b) steel, (c) aluminum foil, (d) ceramic tile, (e) glass, (f) plastic, (g) rubber, (h) leather, (i) cardboard and (j) white paper. Each image was cropped to the area of the mark from the original photograph without further adjustment.

**Fig. 5:**
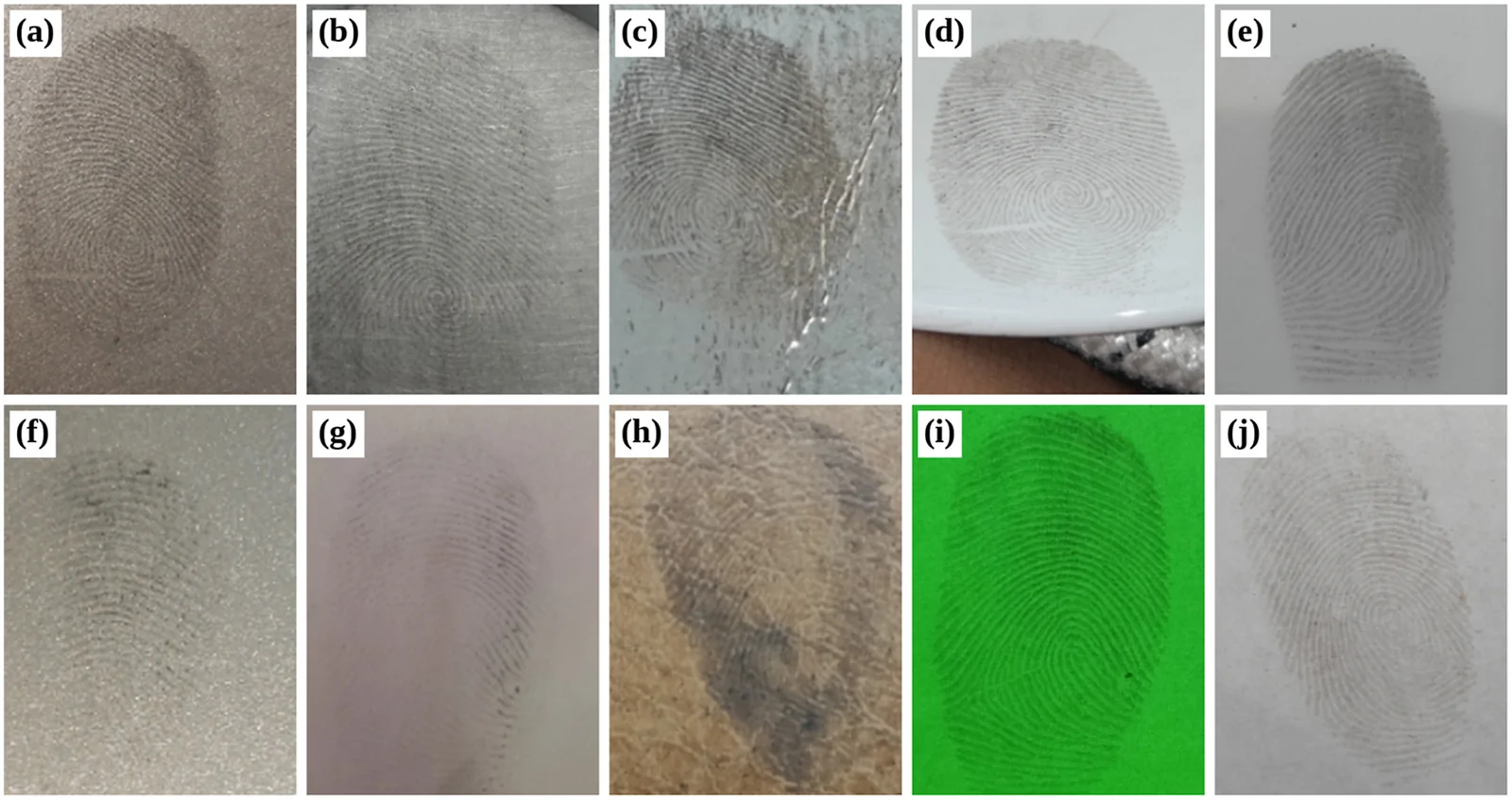
Natural fingermarks developed with MnCo_2_O_4_ powder on (a) iron, (b) steel, (c) aluminum foil, (d) ceramic tile, (e) glass, (f) plastic, (g) rubber, (h) leather, (i) cardboard and (j) white paper. Each image was cropped to the area of the mark from the original photograph without further adjustment.

The EDS spectrum (Fig. 3c) shows signals assigned to Mn, Co, and O, and quantification of these elements gave mass fractions of 26.9 wt% Mn, 50.9 wt% Co, and 22.1 wt% O. Conversion of these values to atomic proportions gives a Co:Mn atomic ratio of approximately 1.8, close to the nominal 2:1 ratio of MnCo_2_O_4_. The measured oxygen fraction is lower than the stoichiometric value; EDS quantification of light elements in rough, particulate specimens is semiquantitative, so this difference alone does not indicate oxygen deficiency [54].

### 3.3 Development of sebaceous fingermarks

MnCo_2_O_4_ powder developed sebaceous marks on all ten substrates (Fig. 4; Table 4). Grade 5 was assigned to iron, steel, ceramic tile, cardboard, and white paper: ridge flow was continuous over the whole mark, minutiae and the pattern area were clearly resolved, and background deposition was negligible. On iron and steel, surface scratches and the reflective metal background did not interrupt the ridge flow (Fig. 4a and b). Grade 4 was assigned to aluminum foil, plastic, and rubber, on which ridge flow was continuous over most of the mark and minutiae were identifiable, with minor discontinuity caused by the crinkled texture of the foil, background deposition along the folds of the plastic, and areas of weak development on the rubber (Fig. 4c–f, g). Grade 3 was assigned to glass and leather, on which ridge flow was visible but interrupted by background interference: on glass, the gray ridges showed low contrast against the transparent substrate, and on leather, powder deposited in the surface grain obscured part of the ridge detail (Fig. 4e–h).

### 3.4 Development of natural fingermarks

Natural marks were developed on all ten substrates and received lower grades than sebaceous marks on six of them (Fig. 5; Table 4). Grade 5 was assigned to ceramic tile and cardboard, with continuous ridge flow over the whole mark and negligible background deposition (Fig. 5d–i). Grade 4 was assigned to iron, aluminum foil, and white paper: ridge flow was continuous over most of the mark, with minor discontinuities caused by the surface texture of the iron and foil, and lower contrast on white paper (Fig. 5a–c, j). Grade 3 was assigned to steel and glass, on which smudging interrupted the ridge flow and, on steel, the brushed metal background reduced contrast (Fig. 5b–e). Grade 2 was assigned to plastic, rubber, and leather, on which ridge development was faint or partial, and ridge continuity was largely lost; on leather, the powder formed a uniform deposit in which individual ridges could not be followed over most of the mark (Fig. 5f, g, h).

### 3.5 Comparison of deposit types and substrate classes

The clarity grades for all 20 substrate and deposit combinations are given in Table 4 and Fig. 6. The mean grade was 4.3 (median 4.5) for sebaceous marks and 3.4 (median 3.5) for natural marks. Eight of ten substrates reached grade 4 or 5 with sebaceous marks and five of ten with natural marks, and no substrate received a grade below 3 for sebaceous marks (Fig. 6c). No substrate gave a higher grade for natural marks than for sebaceous marks. Grades were equal on aluminum foil, ceramic tile, glass, and cardboard, lower by one grade for natural marks on iron, leather, and white paper, and lower by two grades on steel, plastic, and rubber (Fig. 6a and b). The difference between deposit types was statistically significant (Wilcoxon signed-rank test, W = 0, n = 6 nonzero pairs, p = 0.031).

**Fig. 6:**
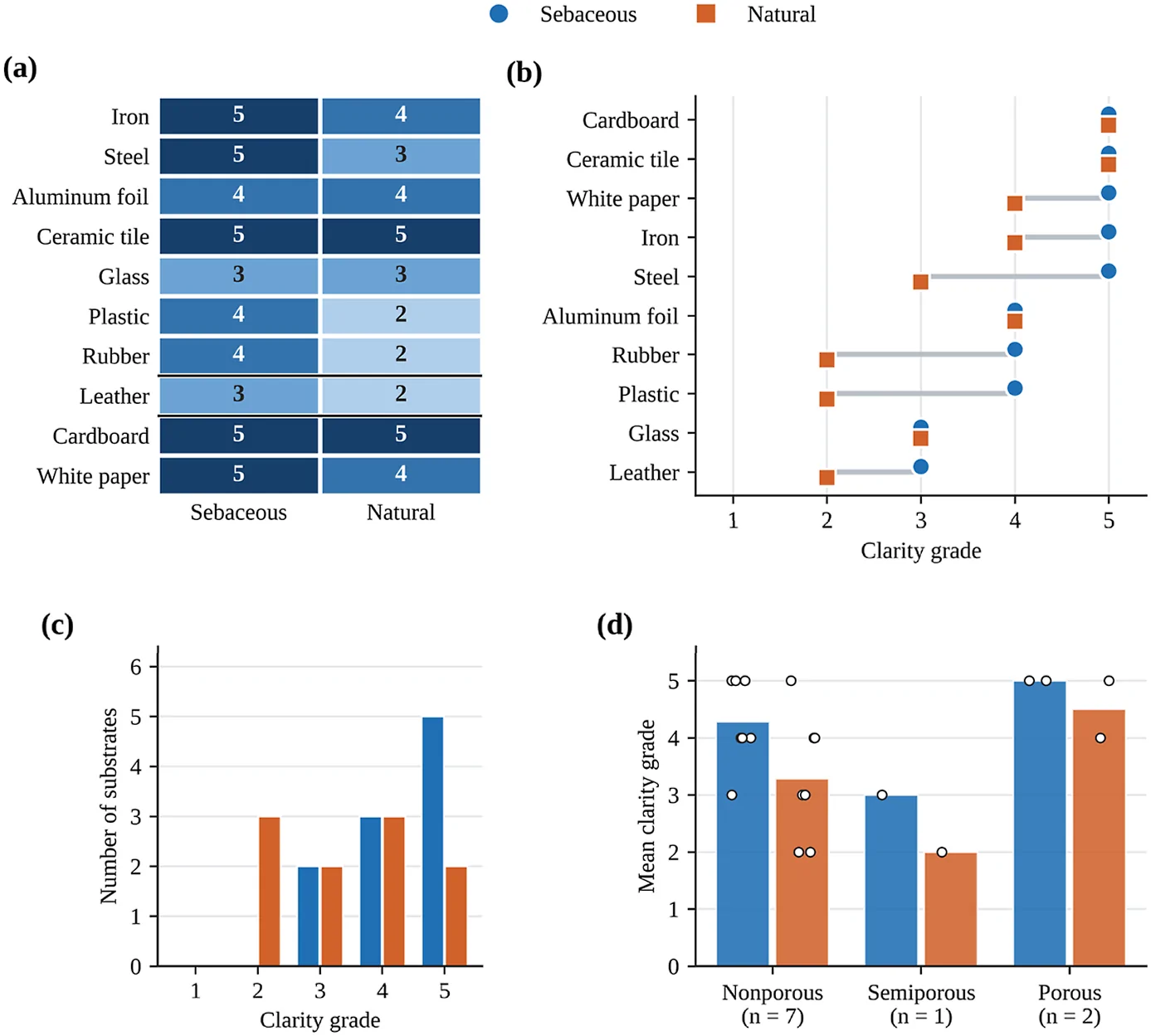
Clarity grades of fingermarks developed with MnCo_2_O_4_. (a) Grade matrix for sebaceous and natural marks on the ten substrates; horizontal lines separate nonporous, semiporous, and porous substrates. (b) Paired grades for each substrate, ordered by combined grade; blue circles denote sebaceous and orange squares denote natural marks. (c) Number of substrates at each grade. (d) Mean grade by porosity class, with individual substrate grades shown as open circles.

By porosity class, nonporous substrates (n = 7) gave mean grades of 4.29 for sebaceous and 3.29 for natural marks, the semiporous leather gave 3 and 2, and the porous substrates (n = 2) gave 5.0 and 4.5 (Fig. 6d). Within the nonporous class, grades ranged from 3 to 5 for sebaceous marks and from 2 to 5 for natural marks, so that substrate texture and reflectance within a class were associated with larger differences than the class itself. The semiporous and porous classes each contain one or two substrates, and their means describe those substrates rather than the classes in general.

### 3.6 Objective image quality

The ridge period of the cropped marks in the source photographs ranged from 3.3 to 7.1 pixels (median 3.85), so normalization to the 500 ppi equivalent required upsampling by factors of 1.3 to 2.8. Without contrast normalization, NFIQ 2 feature extraction failed for 13 of the 20 images, including all ten natural marks. After the global contrast stretch, scores were computed for 19 images; for the natural mark on rubber, the embedded extractor returned an insufficient fingerprint area, and no score could be computed.

NFIQ 2 scores ranged from 22 to 62 for sebaceous marks (mean 35.8, median 33.5) and from 1 to 43 for the nine computable natural marks (mean 20.2, median 15.0) (Table 5; Fig. 7a–d). The highest scores were obtained for sebaceous marks on ceramic tile (62), cardboard (48), and white paper (43) and for the natural mark on cardboard (43); the lowest were obtained for natural marks on plastic (1), leather (10), and aluminum foil (12). Glass was the only substrate on which the natural mark scored marginally higher than the sebaceous mark (31 versus 29). Sebaceous marks scored higher than natural marks across substrates (Wilcoxon signed-rank test, W = 1, n = 10, p = 0.004). NFIQ 2 scores correlated positively with the visual clarity grades (Spearman ρ = 0.69, p = 0.001, n = 19; ρ = 0.74, p < 0.001, n = 20 with the failed image assigned 0) (Fig. 7b). The largest discordances were the sebaceous mark on steel (grade 5, score 22) and the natural mark on white paper (grade 4, score 17), both of which scored lower than their grades suggested, and the natural mark on glass (grade 3, score 31), which scored higher.

**Table 5:** NFIQ 2 scores, minutiae counts (MC) and OCL-based minutiae quality (MQ OCL) of the developed fingermarks.

Substrate	NFIQ 2 Seb.	NFIQ 2 Nat.	MC Seb.	MC Nat.	MQ OCL Seb.	MQ OCL Nat.
Iron	31	15	114	255	0.053	0.000
Steel	22	15	121	148	0.033	0.000
Aluminum foil	36	12	255	255	0.133	0.039
Ceramic tile	62	38	113	27	0.407	0.148
Glass	29	31	103	66	0.068	0.318
Plastic	24	1	119	161	0.143	0.000
Rubber	40	NC	86	NC	0.174	NC
Leather	23	10	255	255	0.000	0.000
Cardboard	48	43	133	75	0.308	0.080
White paper	43	17	93	81	0.462	0.025
Median	33.5	15.0	116.5	148	0.138	0.025

**Fig. 7:**
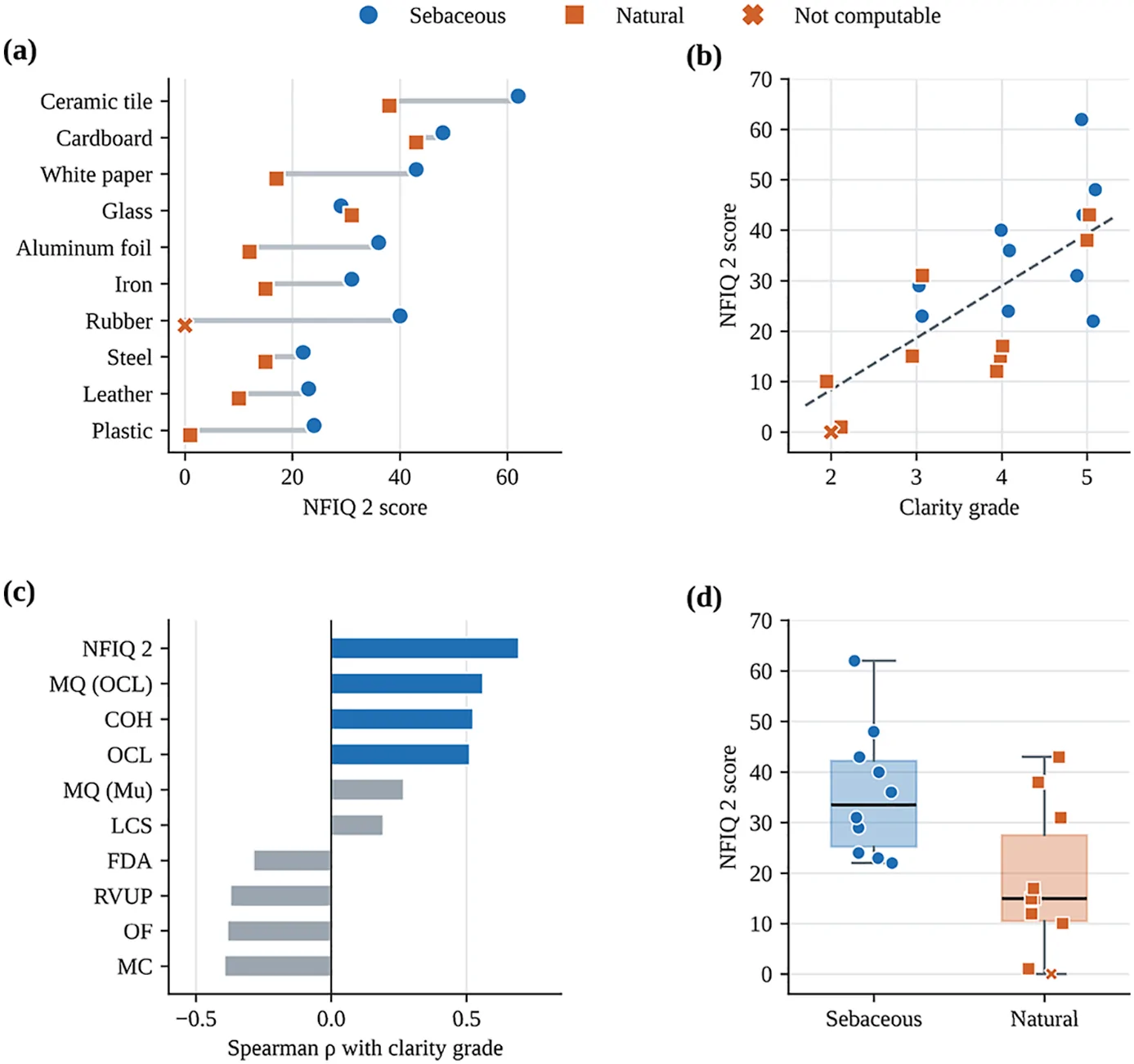
NFIQ 2 assessment of fingermarks developed with MnCo_2_O_4_. (a) Paired NFIQ 2 scores of sebaceous (circles) and natural (squares) marks for each substrate; the cross denotes the natural mark on rubber, for which no score could be computed. (b) NFIQ 2 score against visual clarity grade with a least-squares guideline. (c) Spearman correlation of NFIQ 2 outputs with the clarity grade; blue bars denote p < 0.05. NFIQ 2, unified quality score; MQ, minutiae quality; COH, relative orientation coherence; OCL, orientation certainty level; LCS, local clarity score; FDA, frequency domain analysis; RVUP, ridge valley uniformity; OF, orientation flow; MC, minutiae count. (d) Distribution of NFIQ 2 scores by deposit type.

Among the native quality measures, orientation certainty (OCL; ρ = 0.51, p = 0.025), relative orientation coherence (COH; ρ = 0.53, p = 0.021) and the OCL-based minutiae quality (MQ OCL; ρ = 0.56, p = 0.012) correlated positively with the visual grades (Fig. 7c). Local clarity, frequency domain, ridge valley uniformity and orientation flow measures did not correlate significantly with the grades. The minutiae counts (MC) in Table 5 are the numbers of features returned by the automated extractor over the whole normalized image. They include detections in the substrate texture outside the ridge pattern and are not counts of minutiae verified by an examiner (Section 3.7). The number of minutiae detected by the embedded extractor was inversely, though not significantly, related to the grades (ρ = −0.40, p = 0.094) and reached the extractor limit of 255 minutiae for both marks on aluminum foil and leather and for the natural mark on iron.

### 3.7 Automated minutiae extraction

Minutiae maps produced by the FingerJet FX OSE extractor are shown in Fig. 8. On marks with high NFIQ 2 scores, the sebaceous mark on ceramic tile and the natural mark on cardboard, the detected ridge endings and bifurcations were located within the developed ridge area and followed the ridge flow, with additional detections concentrated along the margins of the mark (Fig. 8a and b). On the sebaceous mark on leather and the natural mark on aluminum foil, a large proportion of the detected minutiae lay in the background grain or in the crinkled texture of the foil, outside the ridge pattern (Fig. 8c and d). These spurious detections account for the extractor limit being reached on these substrates.

**Fig. 8:**
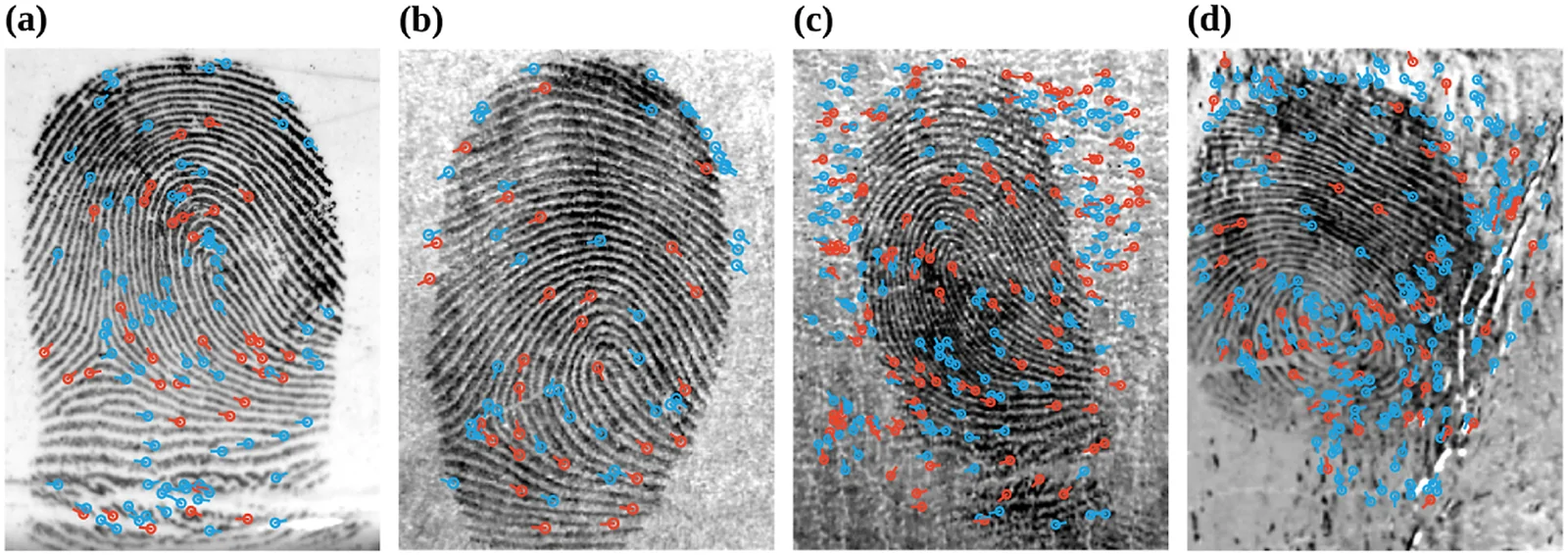
Minutiae detected by the FingerJet FX OSE extractor on normalized images of (a) the sebaceous mark on ceramic tile, (b) the natural mark on cardboard, (c) the sebaceous mark on leather and (d) the natural mark on aluminum foil. Red symbols denote ridge endings and blue symbols denote bifurcations; the tail indicates minutia direction.

### 3.8 Microscopic distribution of the powder on ridges

Under the microscope, ridges developed with MnCo_2_O_4_ powder on glass appeared as bands of densely packed dark particle aggregates that followed the ridge flow, separated by furrows that were largely free of particles. Aggregates of different sizes were present along the ridges, and isolated particles were scattered sparsely between them. In the field developed with Sirchie black powder, the ridges were also defined by particle deposition. However, the deposit was more diffuse at the ridge edges, and fine particles were distributed more widely across the furrows and background. These observations were made on different marks and describe particle distribution in the fields examined; they do not constitute a graded comparison of the two powders.

## 4 Discussion

### 4.1 Relation between powder characteristics and development

Powder development depends on the preferential retention of particles by the ridge deposit relative to the substrate, a process governed by particle size, shape, and surface properties, as well as the physical state of the residue [6], [7]. The MnCo_2_O_4_ powder combined nanocrystalline domains of approximately 21 nm with 50 nm particles that formed larger aggregates, and the micrographs show that the deposited material was retained mainly as aggregates on the ridges. The hydroxyl bands observed by FTIR indicate a hydrated, hydroxylated oxide surface, which may influence wetting by and adhesion to the residue. The present data do not identify the interaction responsible for retention: no measurements of surface energy, zeta potential, or residue binding were made, and the observed selectivity is consistent with the physical adhesion of fine particles to a moist or lipid-rich deposit, as described for conventional powders [2], [6]. The black color of the powder provided contrast on light substrates such as ceramic tile, cardboard, and white paper, where the highest grades were recorded for both deposit types.

The formation of a spinel phase at 80 °C without calcination is consistent with reports that cubic Mn–Co spinel nanocrystals can form from aqueous precursors through precipitation and oxidation at low temperature [35], [36]. Calcination conditions have been shown to influence the properties and electrochemical performance of mesoporous Mn_1−x_Ni_x_Co_2_O_4_ nanoparticles [55], and thermal treatment could be used to adjust the crystallinity of the present powder if required. The weak, broad reflections and the sloping XRD background indicate that the product was not fully crystalline. For a dusting powder, this is not necessarily a disadvantage, because development depends on particle size and surface properties rather than long-range order. Batch-to-batch reproducibility of the phase composition and lattice parameter was not assessed here and would be needed before routine use.

### 4.2 Effect of deposit type

The consistently higher grades for sebaceous marks agree with the greater quantity of lipid material transferred when the fingertips are loaded from the face [3], [4]. The natural marks were not chemically characterized in this study. Because they were deposited without deliberate enrichment or depletion (Section 2.4), they are best regarded as variable mixtures of eccrine and sebaceous residues with a lower and less predictable lipid loading than groomed sebaceous marks, rather than as eccrine-dominant deposits [3], [5], [49]. A larger, more viscous deposit would be expected to retain more powder and tolerate brushing better, which is consistent with the largest differences between deposit types occurring on plastic, rubber, and steel surfaces, on which the adhesion of a thin deposit is weakest. Groomed sebaceous marks represent a favorable condition, and fingermark research guidelines caution that results obtained with them can overstate performance on casework marks [49], [50]. The natural mark grades are the more relevant indicator of performance on casework marks, although they remain laboratory results: under that condition, the powder gave grade 4 or 5 on ceramic tile, cardboard, white paper, iron and aluminum foil, and only grade 2 on rubber, plastic and leather.

### 4.3 Effect of substrate

The ranking of substrates did not follow porosity class alone. Smooth, light-colored surfaces (ceramic tile, cardboard, and white paper) produced the best results, whereas flexible or textured surfaces (rubber, plastic, and leather) produced the poorest natural mark grades. The leather's textured grain retained powder outside the ridges, resulting in background deposition. The low surface energy and reflectance of the plastic and rubber surfaces may have limited both adhesion and photographic contrast; surface energy was not measured, so this explanation remains an interpretation. On glass, the gray appearance of the ridges against the transparent substrate limited contrast for both deposit types, indicating that a contrasting background or alternative lighting would be required to image dark powder on glass. The high grades on cardboard and white paper were obtained for marks developed without a deliberate aging interval. Powders are generally recommended for nonporous surfaces because residue on porous substrates is absorbed into the substrate over time [2], [5], and the performance on porous substrates reported here may not persist for aged marks.

### 4.4 Objective quality assessment

NFIQ 2 provided an observer-independent measure that ranked the developed marks in broad agreement with the visual grades and reproduced the main findings of the visual assessment: higher quality for sebaceous than for natural marks, and the highest quality on ceramic tile and cardboard. The native measures that correlated with the grades, orientation certainty and orientation coherence, describe the continuity and regularity of ridge flow, which is the principal criterion of the clarity scale (Table 2). Absolute NFIQ 2 values were low, with no image above 62. NFIQ 2 was trained on plain impressions captured by optical scanners or ink at 500 ppi [45]. In contrast, the present images were photographs of powdered marks on textured, colored, or reflective substrates, upsampled from a lower native ridge resolution. The scores are interpreted here as relative values within the study, not as predictions of matching performance in an automated identification system. Discordant cases indicate where the two approaches weigh evidence differently: on brushed steel, the reflective background reduced grayscale contrast and orientation certainty, lowering the NFIQ 2 score of a mark that the assessors graded as excellent, because assessors discount such background more readily than an algorithm does.

These observations have three implications for the use of NFIQ 2 in fingermark development research. First, absolute scores of developed marks are not comparable to the quality thresholds applied to reference prints captured under controlled conditions, and a low score for a developed mark does not, by itself, indicate that the mark lacks value for comparison. Second, NFIQ 2 can serve as a reproducible comparator between development techniques, substrates, or deposit types, provided that image acquisition and normalization are fixed and reported; photographs taken with a scale and at a resolution of at least 500 ppi would remove the need for the rescaling based on ridge period that was applied here. Third, the discordances on reflective and textured substrates indicate that NFIQ 2 does not replace examiners' clarity assessment. Approaches developed for latent marks, such as the clarity assessment of friction ridge impressions by examiners [56], latent image quality metrics that predict automated matching performance and correlate with the value determinations of examiners [57], and quantitative image measures that predict the difficulty of fingerprint comparisons for examiners [58], are more directly related to forensic utility. Their application to developed marks remains to be evaluated.

Automated minutiae counts were not a reliable indicator of the quality of development for these images. Background texture on aluminum foil, leather, and iron generated ridge-like structures that the extractor interpreted as minutiae, resulting in the poorest marks yielding the largest counts. Minutiae-based metrics applied to developed marks require segmentation of the ridge area or examiner verification of detected features, a requirement also noted for the automated processing of latent marks in forensic identification workflows [46]. Within these constraints, combining a visual grade with NFIQ 2 scoring adds a reproducible, quantitative layer to the evaluation of a developing powder. It enables results to be compared across laboratories that use the same image normalization.

### 4.5 Comparison with other particulate developers

Several inorganic and naturally derived powders have been evaluated on comparable sets of porous and nonporous substrates, including ZnO, ZnO–SiO_2_ and TiO_2_ nanopowders, metal and metal oxide nanoparticles, iron oxide and cobalt ferrite magnetic powders, and powders derived from plants and waste such as gram flour, jamun seed, onion, pomegranate peel, water chestnut flour, aloe vera and wheatgrass [16], [17], [18], [19], [20], [21], [26], [28], [29], [30], [31], [32], [33], [34]. The surface-specific behavior reported for metal and metal oxide nanoparticles [20] is consistent with the substrate dependence observed here for MnCo_2_O_4_. Differences in donors, deposit types, substrates, aging, and grading scales prevent direct numerical comparison with these studies. Within the present work, the microscopic comparison on glass indicated that MnCo_2_O_4_ powder was retained on the ridges with limited deposition in the furrows. In contrast, the commercial black powder produced a more diffuse deposit in the field examined. A split-mark design, graded on the same marks as recommended for comparative evaluation [50], is required to determine whether this difference translates into greater clarity across substrates.

### 4.6 Handling considerations

MnCo_2_O_4_ contains cobalt and is handled as a fine powder. The International Agency for Research on Cancer (IARC) has classified cobalt metal and soluble cobalt(II) salts as probably carcinogenic to humans [59]. Mixed Mn–Co oxides were not evaluated in that classification, and the toxicity of the present material was not assessed. Until such data are available, application of the powder should follow the precautions used for respirable particulate reagents, including local exhaust ventilation, respiratory protection and avoidance of skin contact.

### 4.7 Scope of the findings

The present results establish the laboratory feasibility of MnCo_2_O_4_ as a dusting powder for fresh sebaceous and natural marks on the ten substrates examined; they do not demonstrate suitability for operational use. Grades and NFIQ 2 scores were obtained for one representative mark per substrate and deposit type, so donor variability is not represented in the statistical comparisons, which rest on paired values from ten substrates. All marks were developed without an aging interval, and eccrine marks and marks exposed to environmental conditions were not examined. The particle size was measured from a single micrograph; the comparison with Sirchie black powder was qualitative and performed on different marks; and batch-to-batch reproducibility of the powder was not assessed. Within the framework of the International Fingerprint Research Group (IFRG), the study constitutes an initial evaluation; optimization, split-mark comparison with established powders, and pseudo-operational testing on a larger set of natural marks are required before casework application can be considered [50].

## 5 Conclusion

MnCo_2_O_4_ nanoparticles with a cubic spinel structure, a crystallite size of approximately 21 nm, and a particle size of 50 nm were obtained by coprecipitation at 80 °C. Under laboratory conditions, the powder developed fresh sebaceous marks to grade 4 or 5 on eight of ten substrates and fresh natural marks to these grades on five substrates, with the best results on ceramic tile, cardboard, and white paper, and the poorest natural mark development on rubber, plastic, and leather. NFIQ 2 scores agreed with the visual grades and were higher for sebaceous marks, whereas automated minutiae counts were inflated by substrate texture. These results establish laboratory feasibility only; aged and eccrine marks, split-mark comparison with commercial powders, pseudo-operational testing and toxicity assessment are required before casework use can be considered.

## CRediT authorship contribution statement

**Shivam Kumar Mittal:** Writing – original draft, Investigation, Formal analysis, Data curation. **Kumud Kant Awasthi:** Project administration, Formal analysis, Conceptualization. **Vaibhav Sharma:** Investigation, Formal analysis, Data curation. **Garima Awasthi:** Writing – review & editing, Visualization, Supervision. **Rachit Manchanda:** Investigation, Formal analysis. **Gaurav Kumar Singh:** Software, Investigation. **Yogesh Kumar:** Software, Investigation. **Harsh Pandey:** Writing – review & editing, Supervision, Resources, Project administration. **Mahipal Singh Sankhla:** Writing – original draft, Supervision, Investigation, Conceptualization.

## Data availability

NFIQ 2 outputs, native quality measures, and image normalization parameters for all developed marks are provided in Supplementary Table S1.

## Declaration of competing interest

The authors declare that they have no known competing financial interests or personal relationships that could have appeared to influence the work reported in this paper.
